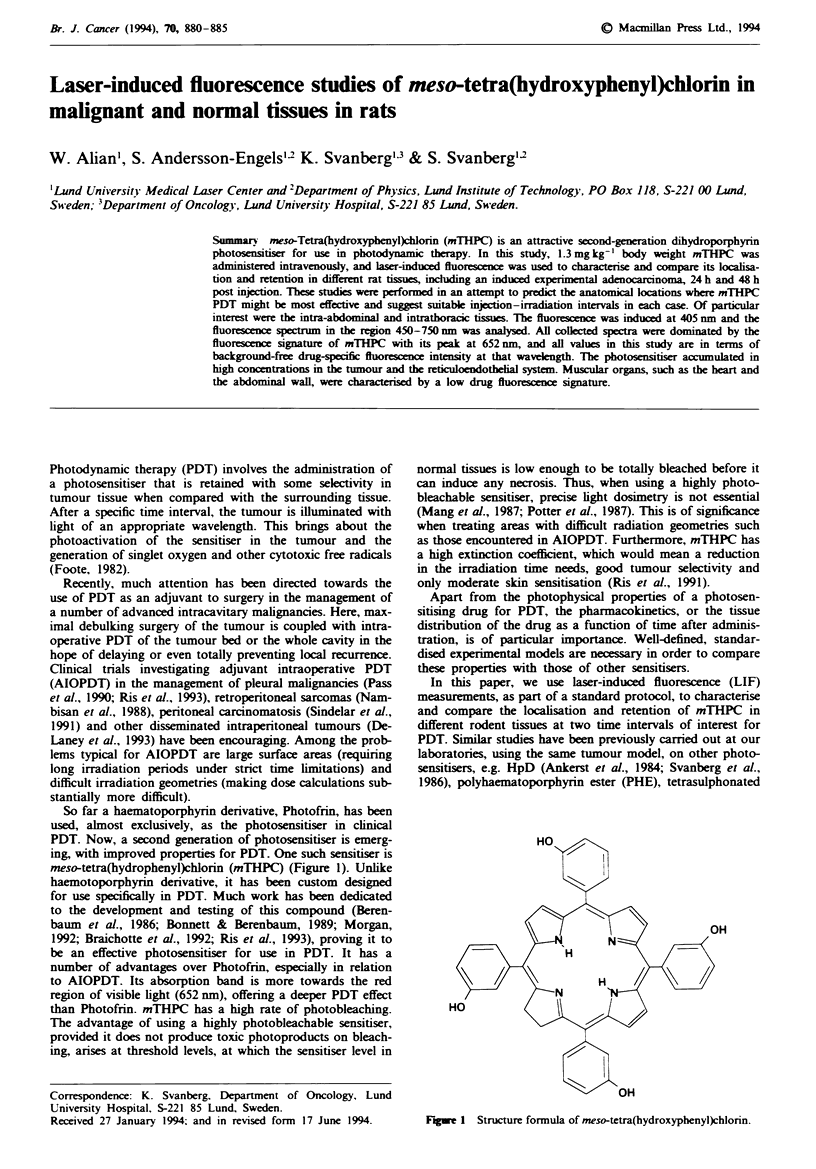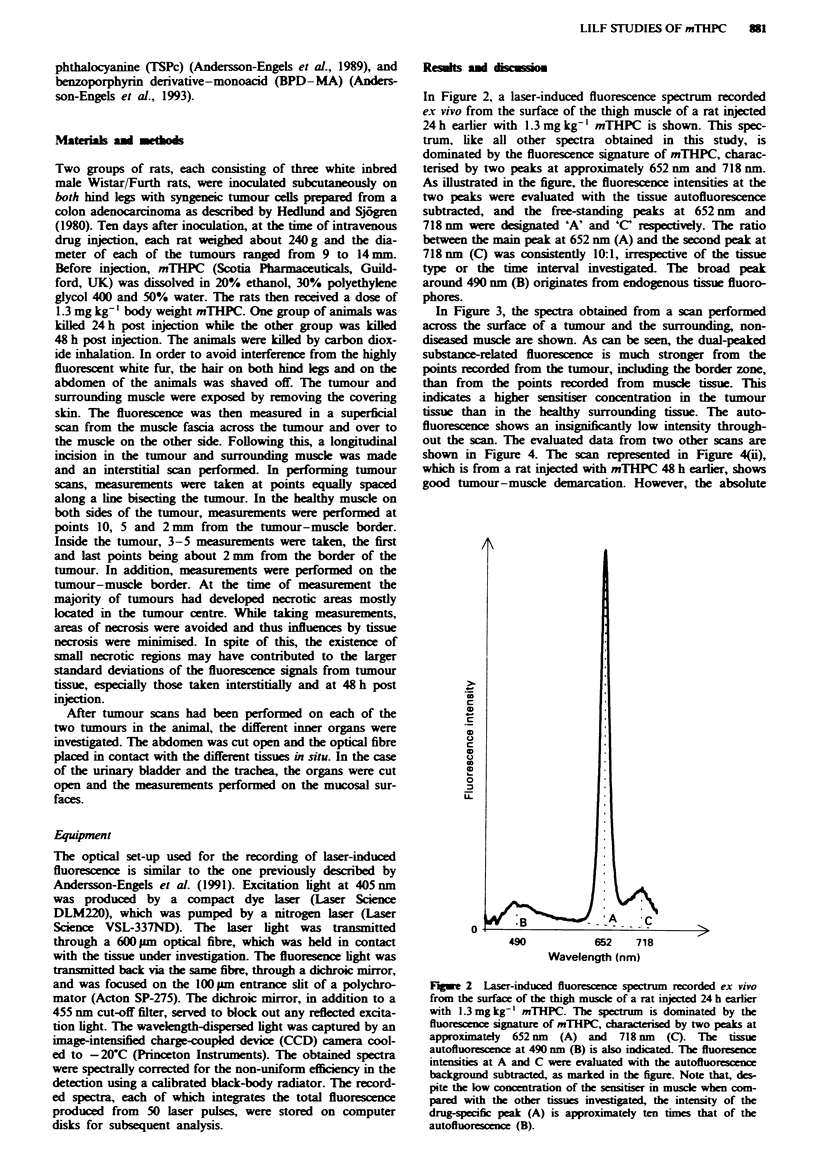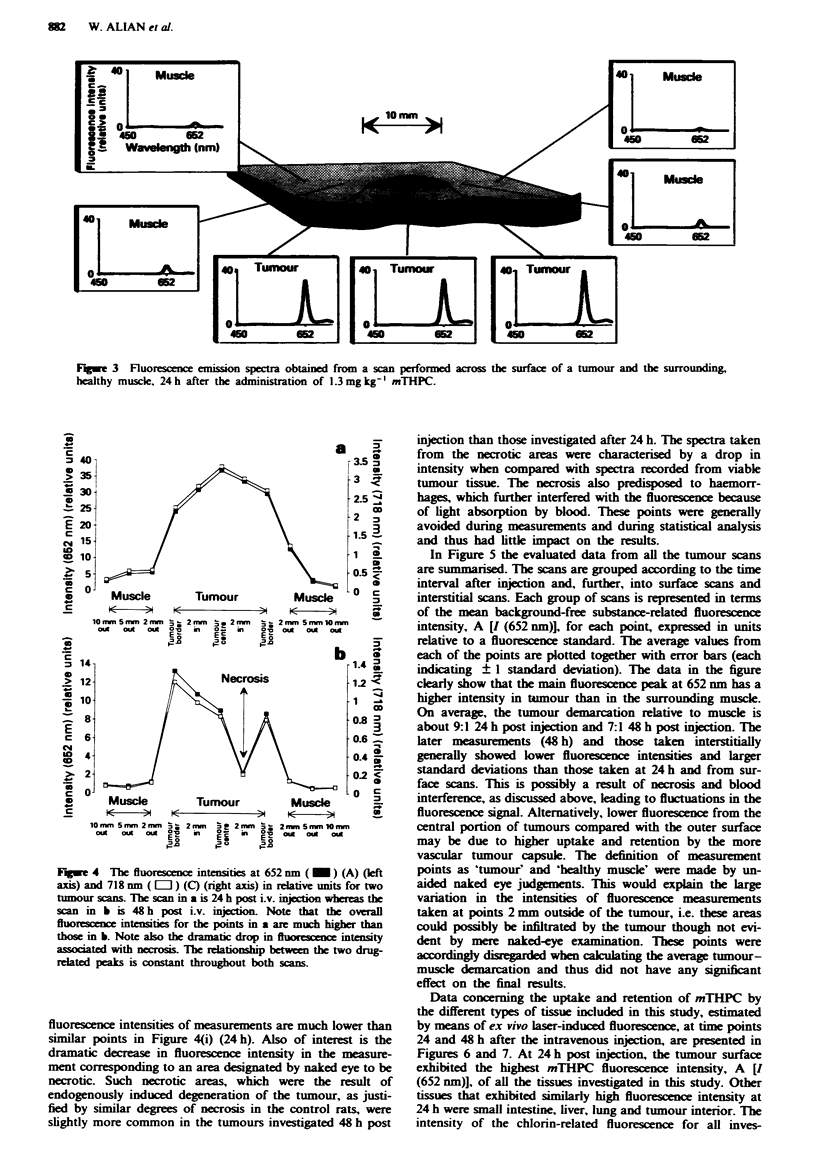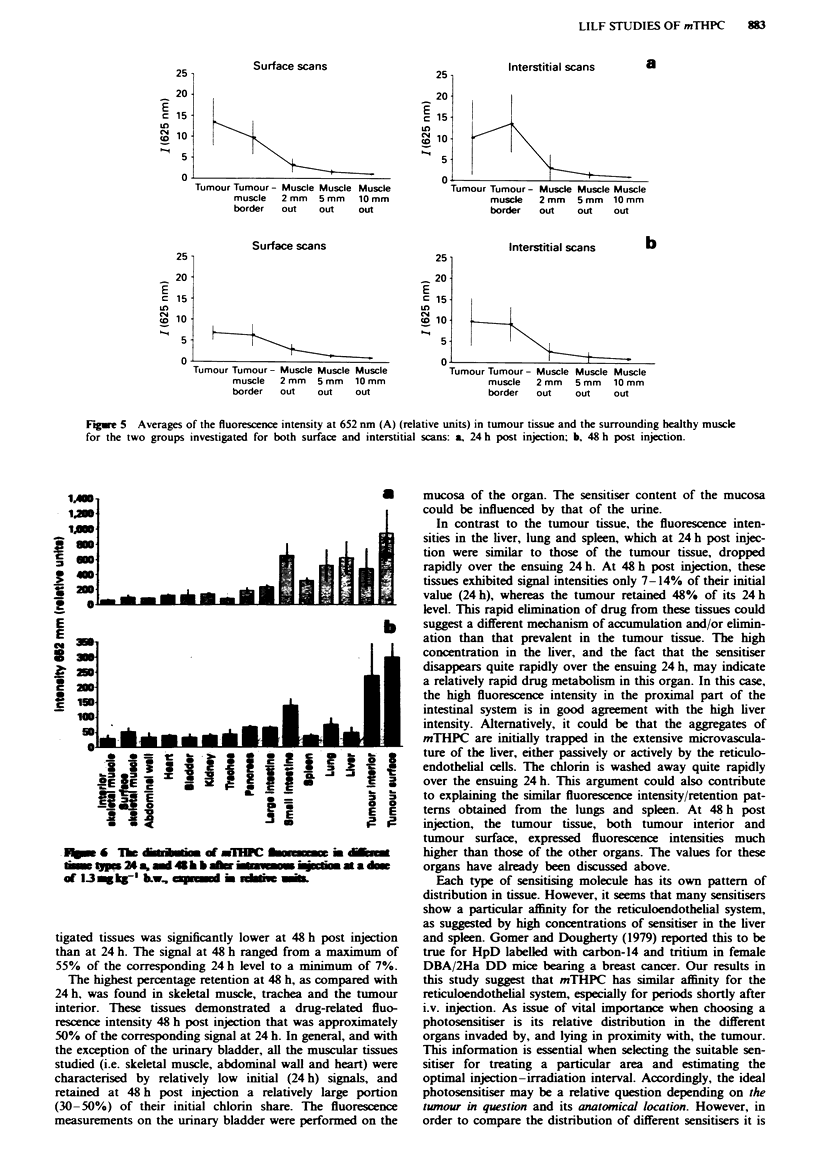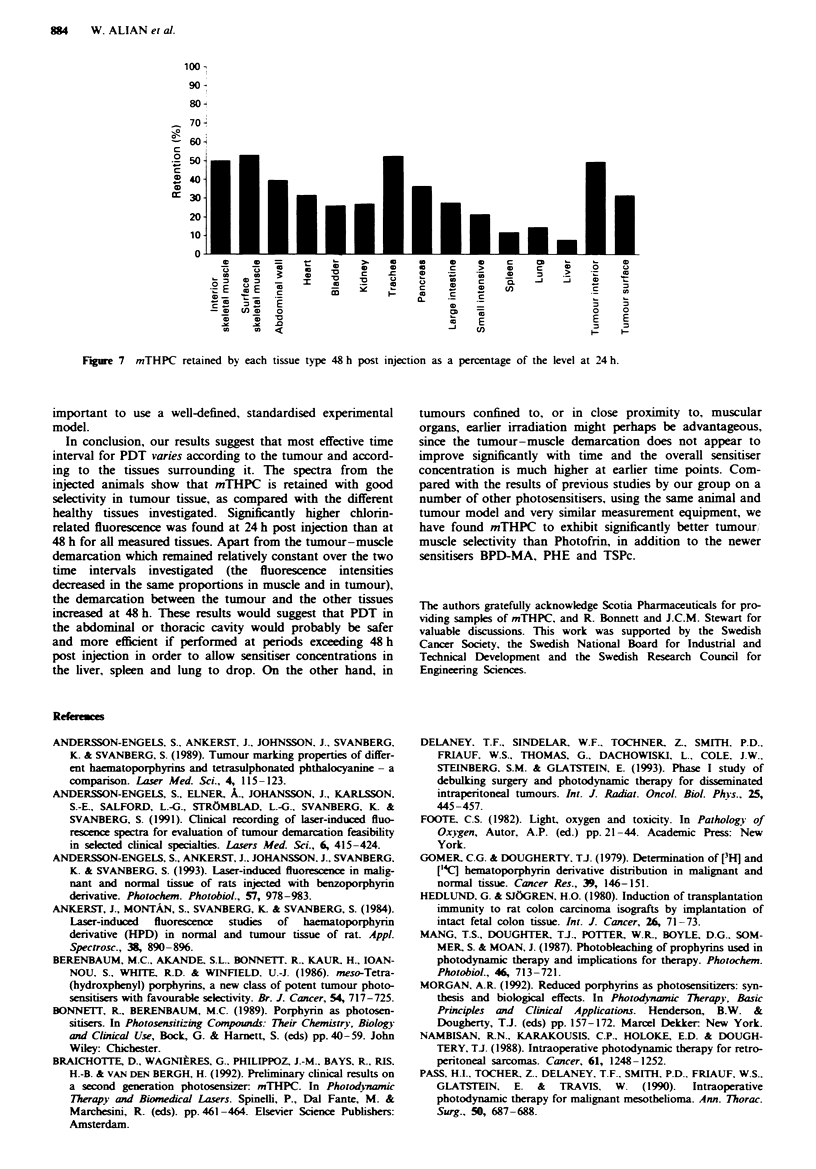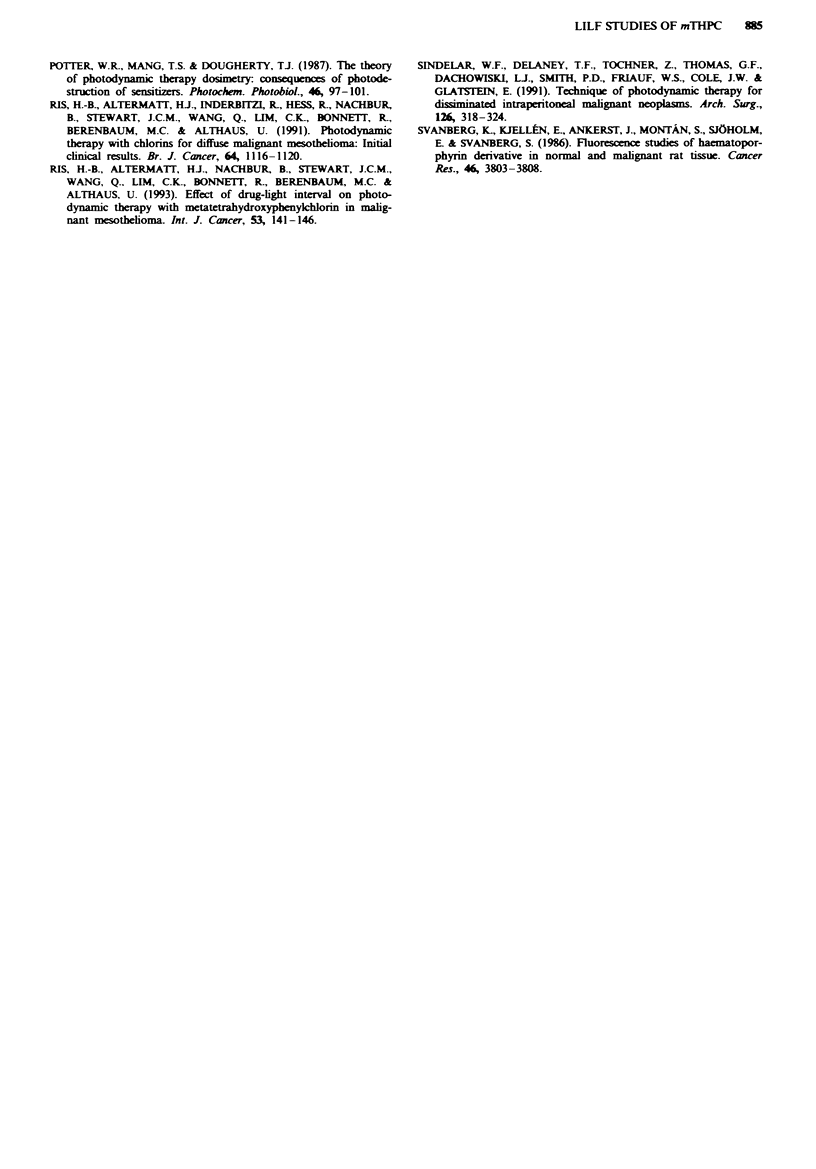# Laser-induced fluorescence studies of meso-tetra(hydroxyphenyl)chlorin in malignant and normal tissues in rats.

**DOI:** 10.1038/bjc.1994.414

**Published:** 1994-11

**Authors:** W. Alian, S. Andersson-Engels, K. Svanberg, S. Svanberg

**Affiliations:** Lund University Medical Laser Center, Sweden.

## Abstract

meso-Tetra(hydroxyphenyl)chlorin (mTHPC) is an attractive second-generation dihydroporphyrin photosensitiser for use in photodynamic therapy. In this study, 1.3 mg kg-1 body weight mTHPC was administered intravenously, and laser-induced fluorescence was used to characterise and compare its localisation and retention in different rat tissues, including an induced experimental adenocarcinoma, 24 h and 48 h post injection. These studies were performed in an attempt to predict the anatomical locations where mTHPC PDT might be most effective and suggest suitable injection--irradiation intervals in each case. Of particular interest were the intra-abdominal and intrathoracic tissues. The fluorescence was induced at 405 nm and the fluorescence spectrum in the region 450-750 nm was analysed. All collected spectra were dominated by the fluorescence signature of mTHPC with its peak at 652 nm, and all values in this study are in terms of background-free drug-specific fluorescence intensity at that wavelength. The photosensitiser accumulated in high concentrations in the tumour and the reticuloendothelial system. Muscular organs, such as the heart and the abdominal wall, were characterised by a low drug fluorescence signature.


*Br. J. Cancer* (1994), **70**, 880–885 © Macmillan Press Ltd., 1994

### Laser-induced fluorescence studies of *meso*-tetra(hydroxyphenyl)chlorin in malignant and normal tissues in rats

W. Alian[1], S. Andersson-Engels[1,2] K. Svanberg[1,3] & S. Svanberg[1,2]

[1]*Lund University Medical Laser Center and* [2]*Department of Physics, Lund Institute of Technology, PO Box 118, S-221 00 Lund, Sweden;* [3]*Department of Oncology, Lund University Hospital, S-221 85 Lund, Sweden.*

**Summary** *meso*-Tetra(hydroxyphenyl)chlorin (*m*THPC) is an attractive second-generation dihydroporphyrin photosensitiser for use in photodynamic therapy. In this study, 1.3 mg $kg^{-1}$ body weight *m*THPC was administered intravenously, and laser-induced fluorescence was used to characterise and compare its localisation and retention in different rat tissues, including an induced experimental adenocarcinoma, 24 h and 48 h post injection. These studies were performed in an attempt to predict the anatomical locations where *m*THPC PDT might be most effective and suggest suitable injection–irradiation intervals in each case. Of particular interest were the intra-abdominal and intrathoracic tissues. The fluorescence was induced at 405 nm and the fluorescence spectrum in the region 450–750 nm was analysed. All collected spectra were dominated by the fluorescence signature of *m*THPC with its peak at 652 nm, and all values in this study are in terms of background-free drug-specific fluorescence intensity at that wavelength. The photosensitiser accumulated in high concentrations in the tumour and the reticuloendothelial system. Muscular organs, such as the heart and the abdominal wall, were characterised by a low drug fluorescence signature.

Photodynamic therapy (PDT) involves the administration of a photosensitiser that is retained with some selectivity in tumour tissue when compared with the surrounding tissue. After a specific time interval, the tumour is illuminated with light of an appropriate wavelength. This brings about the photoactivation of the sensitiser in the tumour and the generation of singlet oxygen and other cytotoxic free radicals (Foote, 1982).

Recently, much attention has been directed towards the use of PDT as an adjuvant to surgery in the management of a number of advanced intracavitary malignancies. Here, maximal debulking surgery of the tumour is coupled with intraoperative PDT of the tumour bed or the whole cavity in the hope of delaying or even totally preventing local recurrence. Clinical trials investigating adjuvant intraoperative PDT (AIOPDT) in the management of pleural malignancies (Pass *et al.*, 1990; Ris *et al.*, 1993), retroperitoneal sarcomas (Nambisan *et al.*, 1988), peritoneal carcinomatosis (Sindelar *et al.*, 1991) and other disseminated intraperitoneal tumours (DeLaney *et al.*, 1993) have been encouraging. Among the problems typical for AIOPDT are large surface areas (requiring long irradiation periods under strict time limitations) and difficult irradiation geometries (making dose calculations substantially more difficult).

So far a haematoporphyrin derivative, Photofrin, has been used, almost exclusively, as the photosensitiser in clinical PDT. Now, a second generation of photosensitiser is emerging, with improved properties for PDT. One such sensitiser is *meso*-tetra(hydrophenyl)chlorin (*m*THPC) (Figure 1). Unlike haemotoporphyrin derivative, it has been custom designed for use specifically in PDT. Much work has been dedicated to the development and testing of this compound (Berenbaum *et al.*, 1986; Bonnett & Berenbaum, 1989; Morgan, 1992; Braichotte *et al.*, 1992; Ris *et al.*, 1993), proving it to be an effective photosensitiser for use in PDT. It has a number of advantages over Photofrin, especially in relation to AIOPDT. Its absorption band is more towards the red region of visible light (652 nm), offering a deeper PDT effect than Photofrin. *m*THPC has a high rate of photobleaching. The advantage of using a highly photobleachable sensitiser, provided it does not produce toxic photoproducts on bleaching, arises at threshold levels, at which the sensitiser level in normal tissues is low enough to be totally bleached before it can induce any necrosis. Thus, when using a highly photobleachable sensitiser, precise light dosimetry is not essential (Mang *et al.*, 1987; Potter *et al.*, 1987). This is of significance when treating areas with difficult radiation geometries such as those encountered in AIOPDT. Furthermore, *m*THPC has a high extinction coefficient, which would mean a reduction in the irradiation time needs, good tumour selectivity and only moderate skin sensitisation (Ris *et al.*, 1991).

Apart from the photophysical properties of a photosensitising drug for PDT, the pharmacokinetics, or the tissue distribution of the drug as a function of time after administration, is of particular importance. Well-defined, standardised experimental models are necessary in order to compare these properties with those of other sensitisers.

In this paper, we use laser-induced fluorescence (LIF) measurements, as part of a standard protocol, to characterise and compare the localisation and retention of *m*THPC in different rodent tissues at two time intervals of interest for PDT. Similar studies have been previously carried out at our laboratories, using the same tumour model, on other photosensitisers, e.g. HpD (Ankerst *et al.*, 1984; Svanberg *et al.*, 1986), polyhaematoporphyrin ester (PHE), tetrasulphonated

**Figure 1** Structure formula of *meso*-tetra(hydroxyphenyl)chlorin.

Correspondence: K. Svanberg, Department of Oncology, Lund University Hospital, S-221 85 Lund, Sweden.
Received 27 January 1994; and in revised form 17 June 1994.

phthalocyanine (TSPc) (Andersson-Engels *et al*., 1989), and benzoporphyrin derivative-monoacid (BPD-MA) (Andersson-Engels *et al*., 1993).

### Materials and methods

Two groups of rats, each consisting of three white inbred male Wistar/Furth rats, were inoculated subcutaneously on *both* hind legs with syngeneic tumour cells prepared from a colon adenocarcinoma as described by Hedlund and Sjögren (1980). Ten days after inoculation, at the time of intravenous drug injection, each rat weighed about 240 g and the diameter of each of the tumours ranged from 9 to 14 mm. Before injection, *m*THPC (Scotia Pharmaceuticals, Guildford, UK) was dissolved in 20% ethanol, 30% polyethylene glycol 400 and 50% water. The rats then received a dose of 1.3 mg $kg^{-1}$ body weight *m*THPC. One group of animals was killed 24 h post injection while the other group was killed 48 h post injection. The animals were killed by carbon dioxide inhalation. In order to avoid interference from the highly fluorescent white fur, the hair on both hind legs and on the abdomen of the animals was shaved off. The tumour and surrounding muscle were exposed by removing the covering skin. The fluorescence was then measured in a superficial scan from the muscle fascia across the tumour and over to the muscle on the other side. Following this, a longitudinal incision in the tumour and surrounding muscle was made and an interstitial scan performed. In performing tumour scans, measurements were taken at points equally spaced along a line bisecting the tumour. In the healthy muscle on both sides of the tumour, measurements were performed at points 10, 5 and 2 mm from the tumour-muscle border. Inside the tumour, 3-5 measurements were taken, the first and last points being about 2 mm from the border of the tumour. In addition, measurements were performed on the tumour-muscle border. At the time of measurement the majority of tumours had developed necrotic areas mostly located in the tumour centre. While taking measurements, areas of necrosis were avoided and thus influences by tissue necrosis were minimised. In spite of this, the existence of small necrotic regions may have contributed to the larger standard deviations of the fluorescence signals from tumour tissue, especially those taken interstitially and at 48 h post injection.

After tumour scans had been performed on each of the two tumours in the animal, the different inner organs were investigated. The abdomen was cut open and the optical fibre placed in contact with the different tissues *in situ*. In the case of the urinary bladder and the trachea, the organs were cut open and the measurements performed on the mucosal surfaces.

### *Equipment*

The optical set-up used for the recording of laser-induced fluorescence is similar to the one previously described by Andersson-Engels *et al*. (1991). Excitation light at 405 nm was produced by a compact dye laser (Laser Science DLM220), which was pumped by a nitrogen laser (Laser Science VSL-337ND). The laser light was transmitted through a 600 μm optical fibre, which was held in contact with the tissue under investigation. The fluoresence light was transmitted back via the same fibre, through a dichroic mirror, and was focused on the 100 μm entrance slit of a polychromator (Acton SP-275). The dichroic mirror, in addition to a 455 nm cut-off filter, served to block out any reflected excitation light. The wavelength-dispersed light was captured by an image-intensified charge-coupled device (CCD) camera cooled to −20°C (Princeton Instruments). The obtained spectra were spectrally corrected for the non-uniform efficiency in the detection using a calibrated black-body radiator. The recorded spectra, each of which integrates the total fluorescence produced from 50 laser pulses, were stored on computer disks for subsequent analysis.

### Results and discussion

In Figure 2, a laser-induced fluorescence spectrum recorded *ex vivo* from the surface of the thigh muscle of a rat injected 24 h earlier with 1.3 mg $kg^{-1}$ *m*THPC is shown. This spectrum, like all other spectra obtained in this study, is dominated by the fluorescence signature of *m*THPC, characterised by two peaks at approximately 652 nm and 718 nm. As illustrated in the figure, the fluorescence intensities at the two peaks were evaluated with the tissue autofluorescence subtracted, and the free-standing peaks at 652 nm and 718 nm were designated 'A' and 'C' respectively. The ratio between the main peak at 652 nm (A) and the second peak at 718 nm (C) was consistently 10:1, irrespective of the tissue type or the time interval investigated. The broad peak around 490 nm (B) originates from endogenous tissue fluorophores.

In Figure 3, the spectra obtained from a scan performed across the surface of a tumour and the surrounding, non-diseased muscle are shown. As can be seen, the dual-peaked substance-related fluorescence is much stronger from the points recorded from the tumour, including the border zone, than from the points recorded from muscle tissue. This indicates a higher sensitiser concentration in the tumour tissue than in the healthy surrounding tissue. The autofluorescence shows an insignificantly low intensity throughout the scan. The evaluated data from two other scans are shown in Figure 4. The scan represented in Figure 4(ii), which is from a rat injected with *m*THPC 48 h earlier, shows good tumour-muscle demarcation. However, the absolute

**Figure 2** Laser-induced fluorescence spectrum recorded *ex vivo* from the surface of the thigh muscle of a rat injected 24 h earlier with 1.3 mg $kg^{-1}$ *m*THPC. The spectrum is dominated by the fluorescence signature of *m*THPC, characterised by two peaks at approximately 652 nm (A) and 718 nm (C). The tissue autofluorescence at 490 nm (B) is also indicated. The fluoresence intensities at A and C were evaluated with the autofluorescence background subtracted, as marked in the figure. Note that, despite the low concentration of the sensitiser in muscle when compared with the other tissues investigated, the intensity of the drug-specific peak (A) is approximately ten times that of the autofluorescence (B).

**Figure 3** Fluorescence emission spectra obtained from a scan performed across the surface of a tumour and the surrounding, healthy muscle, 24 h after the administration of 1.3 mg kg$^{-1}$ *m*THPC.

**Figure 4** The fluorescence intensities at 652 nm ( ■ ) (A) (left axis) and 718 nm ( □ ) (C) (right axis) in relative units for two tumour scans. The scan in **a** is 24 h post i.v. injection whereas the scan in **b** is 48 h post i.v. injection. Note that the overall fluorescence intensities for the points in **a** are much higher than those in **b**. Note also the dramatic drop in fluorescence intensity associated with necrosis. The relationship between the two drug-related peaks is constant throughout both scans.

fluorescence intensities of measurements are much lower than similar points in Figure 4(i) (24 h). Also of interest is the dramatic decrease in fluorescence intensity in the measurement corresponding to an area designated by naked eye to be necrotic. Such necrotic areas, which were the result of endogenously induced degeneration of the tumour, as justified by similar degrees of necrosis in the control rats, were slightly more common in the tumours investigated 48 h post injection than those investigated after 24 h. The spectra taken from the necrotic areas were characterised by a drop in intensity when compared with spectra recorded from viable tumour tissue. The necrosis also predisposed to haemorrhages, which further interfered with the fluorescence because of light absorption by blood. These points were generally avoided during measurements and during statistical analysis and thus had little impact on the results.

In Figure 5 the evaluated data from all the tumour scans are summarised. The scans are grouped according to the time interval after injection and, further, into surface scans and interstitial scans. Each group of scans is represented in terms of the mean background-free substance-related fluorescence intensity, A [*I* (652 nm)], for each point, expressed in units relative to a fluorescence standard. The average values from each of the points are plotted together with error bars (each indicating $\pm$ 1 standard deviation). The data in the figure clearly show that the main fluorescence peak at 652 nm has a higher intensity in tumour than in the surrounding muscle. On average, the tumour demarcation relative to muscle is about 9:1 24 h post injection and 7:1 48 h post injection. The later measurements (48 h) and those taken interstitially generally showed lower fluorescence intensities and larger standard deviations than those taken at 24 h and from surface scans. This is possibly a result of necrosis and blood interference, as discussed above, leading to fluctuations in the fluorescence signal. Alternatively, lower fluorescence from the central portion of tumours compared with the outer surface may be due to higher uptake and retention by the more vascular tumour capsule. The definition of measurement points as 'tumour' and 'healthy muscle' were made by unaided naked eye judgements. This would explain the large variation in the intensities of fluorescence measurements taken at points 2 mm outside of the tumour, i.e. these areas could possibly be infiltrated by the tumour though not evident by mere naked-eye examination. These points were accordingly disregarded when calculating the average tumour–muscle demarcation and thus did not have any significant effect on the final results.

Data concerning the uptake and retention of *m*THPC by the different types of tissue included in this study, estimated by means of *ex vivo* laser-induced fluorescence, at time points 24 and 48 h after the intravenous injection, are presented in Figures 6 and 7. At 24 h post injection, the tumour surface exhibited the highest *m*THPC fluorescence intensity, A [*I* (652 nm)], of all the tissues investigated in this study. Other tissues that exhibited similarly high fluorescence intensity at 24 h were small intestine, liver, lung and tumour interior. The intensity of the chlorin-related fluorescence for all inves-

**Figure 5** Averages of the fluorescence intensity at 652 nm (A) (relative units) in tumour tissue and the surrounding healthy muscle for the two groups investigated for both surface and interstitial scans: **a**, 24 h post injection; **b**, 48 h post injection.

**Figure 6** The distribution of *m*THPC fluorescence in different tissue types 24 **a**, and 48 h **b** after intravenous injection at a dose of 1.3 mg $kg^{-1}$ b.w., expressed in relative units.

tigated tissues was significantly lower at 48 h post injection than at 24 h. The signal at 48 h ranged from a maximum of 55% of the corresponding 24 h level to a minimum of 7%.

The highest percentage retention at 48 h, as compared with 24 h, was found in skeletal muscle, trachea and the tumour interior. These tissues demonstrated a drug-related fluorescence intensity 48 h post injection that was approximately 50% of the corresponding signal at 24 h. In general, and with the exception of the urinary bladder, all the muscular tissues studied (i.e. skeletal muscle, abdominal wall and heart) were characterised by relatively low initial (24 h) signals, and retained at 48 h post injection a relatively large portion (30–50%) of their initial chlorin share. The fluorescence measurements on the urinary bladder were performed on the mucosa of the organ. The sensitiser content of the mucosa could be influenced by that of the urine.

In contrast to the tumour tissue, the fluorescence intensities in the liver, lung and spleen, which at 24 h post injection were similar to those of the tumour tissue, dropped rapidly over the ensuing 24 h. At 48 h post injection, these tissues exhibited signal intensities only 7–14% of their initial value (24 h), whereas the tumour retained 48% of its 24 h level. This rapid elimination of drug from these tissues could suggest a different mechanism of accumulation and/or elimination than that prevalent in the tumour tissue. The high concentration in the liver, and the fact that the sensitiser disappears quite rapidly over the ensuing 24 h, may indicate a relatively rapid drug metabolism in this organ. In this case, the high fluorescence intensity in the proximal part of the intestinal system is in good agreement with the high liver intensity. Alternatively, it could be that the aggregates of *m*THPC are initially trapped in the extensive microvasculature of the liver, either passively or actively by the reticuloendothelial cells. The chlorin is washed away quite rapidly over the ensuing 24 h. This argument could also contribute to explaining the similar fluorescence intensity/retention patterns obtained from the lungs and spleen. At 48 h post injection, the tumour tissue, both tumour interior and tumour surface, expressed fluorescence intensities much higher than those of the other organs. The values for these organs have already been discussed above.

Each type of sensitising molecule has its own pattern of distribution in tissue. However, it seems that many sensitisers show a particular affinity for the reticuloendothelial system, as suggested by high concentrations of sensitiser in the liver and spleen. Gomer and Dougherty (1979) reported this to be true for HpD labelled with carbon-14 and tritium in female DBA/2Ha DD mice bearing a breast cancer. Our results in this study suggest that *m*THPC has similar affinity for the reticuloendothelial system, especially for periods shortly after i.v. injection. As issue of vital importance when choosing a photosensitiser is its relative distribution in the different organs invaded by, and lying in proximity with, the tumour. This information is essential when selecting the suitable sensitiser for treating a particular area and estimating the optimal injection–irradiation interval. Accordingly, the ideal photosensitiser may be a relative question depending on *the tumour in question* and its *anatomical location*. However, in order to compare the distribution of different sensitisers it is

**Figure 7** *m*THPC retained by each tissue type 48 h post injection as a percentage of the level at 24 h.

important to use a well-defined, standardised experimental model.

In conclusion, our results suggest that most effective time interval for PDT *varies* according to the tumour and according to the tissues surrounding it. The spectra from the injected animals show that *m*THPC is retained with good selectivity in tumour tissue, as compared with the different healthy tissues investigated. Significantly higher chlorin-related fluorescence was found at 24 h post injection than at 48 h for all measured tissues. Apart from the tumour-muscle demarcation which remained relatively constant over the two time intervals investigated (the fluorescence intensities decreased in the same proportions in muscle and in tumour), the demarcation between the tumour and the other tissues increased at 48 h. These results would suggest that PDT in the abdominal or thoracic cavity would probably be safer and more efficient if performed at periods exceeding 48 h post injection in order to allow sensitiser concentrations in the liver, spleen and lung to drop. On the other hand, in tumours confined to, or in close proximity to, muscular organs, earlier irradiation might perhaps be advantageous, since the tumour-muscle demarcation does not appear to improve significantly with time and the overall sensitiser concentration is much higher at earlier time points. Compared with the results of previous studies by our group on a number of other photosensitisers, using the same animal and tumour model and very similar measurement equipment, we have found *m*THPC to exhibit significantly better tumour/muscle selectivity than Photofrin, in addition to the newer sensitisers BPD-MA, PHE and TSPc.

The authors gratefully acknowledge Scotia Pharmaceuticals for providing samples of *m*THPC, and R. Bonnett and J.C.M. Stewart for valuable discussions. This work was supported by the Swedish Cancer Society, the Swedish National Board for Industrial and Technical Development and the Swedish Research Council for Engineering Sciences.

### References

ANDERSSON-ENGELS, S., ANKERST, J., JOHNSSON, J., SVANBERG, K. & SVANBERG, S. (1989). Tumour marking properties of different haematoporphyrins and tetrasulphonated phthalocyanine – a comparison. *Laser Med. Sci.*, **4**, 115–123.

ANDERSSON-ENGELS, S., ELNER, Å., JOHANSSON, J., KARLSSON, S.-E., SALFORD, L.-G., STRÖMBLAD, L.-G., SVANBERG, K. & SVANBERG, S. (1991). Clinical recording of laser-induced fluorescence spectra for evaluation of tumour demarcation feasibility in selected clinical specialties. *Lasers Med. Sci.*, **6**, 415–424.

ANDERSSON-ENGELS, S., ANKERST, J., JOHANSSON, J., SVANBERG, K. & SVANBERG, S. (1993). Laser-induced fluorescence in malignant and normal tissue of rats injected with benzoporphyrin derivative. *Photochem. Photobiol.*, **57**, 978–983.

ANKERST, J., MONTÁN, S., SVANBERG, K. & SVANBERG, S. (1984). Laser-induced fluorescence studies of haematoporphyrin derivative (HPD) in normal and tumour tissue of rat. *Appl. Spectrosc.*, **38**, 890–896.

BERENBAUM, M.C., AKANDE, S.L., BONNETT, R., KAUR, H., IOANNOU, S., WHITE, R.D. & WINFIELD, U.-J. (1986). *meso*-Tetra-(hydroxphenyl) porphyrins, a new class of potent tumour photosensitisers with favourable selectivity. *Br. J. Cancer*, **54**, 717–725.

BONNETT, R., BERENBAUM, M.C. (1989). Porphyrin as photosensitisers. In *Photosensitizing Compounds: Their Chemistry, Biology and Clinical Use*, Bock, G. & Harnett, S. (eds) pp. 40–59. John Wiley: Chichester.

BRAICHOTTE, D., WAGNIÈRES, G., PHILIPPOZ, J.-M., BAYS, R., RIS, H.-B. & VAN DEN BERGH, H. (1992). Preliminary clinical results on a second generation photosensizer: *m*THPC. In *Photodynamic Therapy and Biomedical Lasers*. Spinelli, P., Dal Fante, M. & Marchesini, R. (eds). pp. 461–464. Elsevier Science Publishers: Amsterdam.

DELANEY, T.F., SINDELAR, W.F., TOCHNER, Z., SMITH, P.D., FRIAUF, W.S., THOMAS, G., DACHOWISKI, L., COLE, J.W., STEINBERG, S.M. & GLATSTEIN, E. (1993). Phase I study of debulking surgery and photodynamic therapy for disseminated intraperitoneal tumours. *Int. J. Radiat. Oncol. Biol. Phys.*, **25**, 445–457.

FOOTE, C.S. (1982). Light, oxygen and toxicity. In *Pathology of Oxygen*, Autor, A.P. (ed.) pp. 21–44. Academic Press: New York.

GOMER, C.G. & DOUGHERTY, T.J. (1979). Determination of [$^3$H] and [$^{14}$C] hematoporphyrin derivative distribution in malignant and normal tissue. *Cancer Res.*, **39**, 146–151.

HEDLUND, G. & SJÖGREN, H.O. (1980). Induction of transplantation immunity to rat colon carcinoma isografts by implantation of intact fetal colon tissue. *Int. J. Cancer*, **26**, 71–73.

MANG, T.S., DOUGHTER, T.J., POTTER, W.R., BOYLE, D.G., SOMMER, S. & MOAN, J. (1987). Photobleaching of prophyrins used in photodynamic therapy and implications for therapy. *Photochem. Photobiol.*, **46**, 713–721.

MORGAN, A.R. (1992). Reduced porphyrins as photosensitizers: synthesis and biological effects. In *Photodynamic Therapy, Basic Principles and Clinical Applications*. Henderson, B.W. & Dougherty, T.J. (eds) pp. 157–172. Marcel Dekker: New York.

NAMBISAN, R.N., KARAKOUSIS, C.P., HOLOKE, E.D. & DOUGHTERY, T.J. (1988). Intraoperative photodynamic therapy for retroperitoneal sarcomas. *Cancer*, **61**, 1248–1252.

PASS, H.I., TOCHER, Z., DELANEY, T.F., SMITH, P.D., FRIAUF, W.S., GLATSTEIN, E. & TRAVIS, W. (1990). Intraoperative photodynamic therapy for malignant mesothelioma. *Ann. Thorac. Surg.*, **50**, 687–688.

POTTER, W.R., MANG, T.S. & DOUGHERTY, T.J. (1987). The theory of photodynamic therapy dosimetry: consequences of photodestruction of sensitizers. *Photochem. Photobiol.*, **46**, 97–101.

RIS, H.-B., ALTERMATT, H.J., INDERBITZI, R., HESS, R., NACHBUR, B., STEWART, J.C.M., WANG, Q., LIM, C.K., BONNETT, R., BERENBAUM, M.C. & ALTHAUS, U. (1991). Photodynamic therapy with chlorins for diffuse malignant mesothelioma: Initial clinical results. *Br. J. Cancer*, **64**, 1116–1120.

RIS, H.-B., ALTERMATT, H.J., NACHBUR, B., STEWART, J.C.M., WANG, Q., LIM, C.K., BONNETT, R., BERENBAUM, M.C. & ALTHAUS, U. (1993). Effect of drug-light interval on photodynamic therapy with metatetrahydroxyphenylchlorin in malignant mesothelioma. *Int. J. Cancer*, **53**, 141–146.

SINDELAR, W.F., DELANEY, T.F., TOCHNER, Z., THOMAS, G.F., DACHOWISKI, L.J., SMITH, P.D., FRIAUF, W.S., COLE, J.W. & GLATSTEIN, E. (1991). Technique of photodynamic therapy for dissiminated intraperitoneal malignant neoplasms. *Arch. Surg.*, **126**, 318–324.

SVANBERG, K., KJELLÉN, E., ANKERST, J., MONTÁN, S., SJÖHOLM, E. & SVANBERG, S. (1986). Fluorescence studies of haematoporphyrin derivative in normal and malignant rat tissue. *Cancer Res.*, **46**, 3803–3808.